# Be Kind to Yourself: the Implications of Momentary Self-Compassion for Affective Dynamics and Well-Being in Daily Life

**DOI:** 10.1007/s12671-022-02050-y

**Published:** 2023-01-07

**Authors:** Lara Kristin Mey, Mario Wenzel, Karolina Morello, Zarah Rowland, Thomas Kubiak, Oliver Tüscher

**Affiliations:** 1 Leibniz Institute for Resilience Research, Wallstr. 7, 55122 Mainz, Germany; 2 Health Psychology, Institute for Psychology, Johannes Gutenberg University, Binger Str. 14-16, 55122 Mainz, Germany; 3 Department of Psychiatry and Psychotherapy, University Medical Center of the Johannes Gutenberg University, Untere Zahlbacher Strasse 8, 55131 Mainz, Germany

**Keywords:** Self-compassion, Momentary affect, Affective dynamics, Emotional inertia, Stress reactivity, Ecological Momentary Assessment

## Abstract

**Objectives:**

While self-compassion (SC) has mostly been understood as a stable trait-like property, growing evidence suggests that it may fluctuate over time within a given individual. However, little is known on how these fluctuations relate to affective well-being and affective dynamics, such as emotional inertia and stress reactivity in daily life.

**Methods:**

A sample of 119 non-clinical individuals (mean age: 31.3 years, 53.8% female) completed a 7-day smartphone-based ecological momentary assessment study with six semi-random signals per day. With each signal, individuals reported their momentary positive affect (PA) and negative affect (NA), recent SC, and occurrence and perceived strain of daily hassles since the last signal.

**Results:**

Whenever individuals reported higher recent SC than usual, they experienced higher momentary PA and lower momentary NA. Moreover, higher recent SC related to lower stress reactivity in terms of lower decrease of PA and lower increase of NA following the experience of daily hassles. No associations between SC and emotional inertia were found. When distinguishing between the positive components (SC-Pos) and negative components (SC-Neg) of SC, SC-Neg (compared to SC-Pos) was more strongly connected to NA, while SC-Pos and SC-Neg were similarly connected to PA. SC-Pos was associated with an attenuated NA stress reactivity, and SC-Neg with an increased NA stress reactivity. SC-Pos and SC-Neg did not significantly moderate PA stress reactivity nor emotional inertia.

**Conclusions:**

Results show that the benefits of SC for well-being and stress reactivity may unfold whenever we treat ourselves with compassion, irrespective of how self-compassionate we are in general.

**Supplementary Information:**

The online version contains supplementary material available at 10.1007/s12671-022-02050-y.

Self-compassion (SC) describes a “healthy way of relating to oneself in times of suffering” (Neff et al., 2021, p. 1). According to its most common definition by Neff (2003b), SC entails three interacting dimensions with a positive and a negative facet each. First, being self-compassionate means treating oneself with kindness and caring rather than being harsh, criticizing, or judgmental toward oneself (self-kindness vs. self-judgment). Second, it includes the understanding that all our wanted and unwanted experiences are part of human nature, which connect (rather than isolate) us as human beings (common humanity vs. isolation). Last, SC involves an accepting and balanced awareness towards the thoughts, feelings, and circumstances connected to our suffering, instead of being carried away by them (mindfulness vs. over-identification). SC shows strong and consistent connections to the overall well-being (see Zessin et al., 2015, for a meta-analysis) as well as other related psychological outcomes, such as lower levels of depression or anxiety (e.g., Bakker et al., 2019; Neff, 2003a), higher self-esteem (e.g., Krieger et al., 2015; Neff, 2003a), more happiness and optimism (Neff et al., 2007), or higher life-satisfaction (Neff, 2003a).

So far, most studies in the field have conceptualized SC as a stable, trait-like property and mostly assessed it at a single time-point. However, there is growing evidence suggesting that an individual’s SC is not static but may change over time. For instance, levels of SC can be increased through a wide range of SC-based interventions (Ferrari et al., 2019), which either encompass several weekly sessions (e.g., Bluth & Eisenlohr-Moul, 2017; Neff & Germer, 2013) or may be brief, one-session interventions (e.g., Kirschner et al., 2019; Neff et al., 2021). Importantly, in these studies, increases in SC were associated with improvements in a range of trait-oriented outcomes connected to well-being such as lower anxiety (e.g., Neff & Germer, 2013), lower perceived stress (Bluth & Eisenlohr-Moul, 2017; Neff & Germer, 2013), or higher life satisfaction and happiness (Neff & Germer, 2013). Regarding momentary outcomes, it has been found that brief SC interventions led to increases in momentary positive affect (PA) and decreases in momentary negative affect (NA; e.g., Neff et al., 2021) as well as decreases in depressed mood (Diedrich et al., 2014). Hence, SC seems to be a trainable skill rather than a static personality trait.

In addition, a few non-interventional ambulatory studies have conceptualized SC as a state varying across time using daily diary or ecological momentary assessment (EMA) methods, finding important implications for well-being. For instance, daily diary studies found that, on days when individuals reported higher appearance-related or global SC than usual, they indicated healthier eating behaviors (e.g., Breines et al., 2014; Kelly & Stephen, 2016), a better body image (Kelly & Stephen, 2016), less perceived stress (Li et al., 2020), and, overall, a lower probability for clinical impairment due to pathological eating habits compared to other days (Katan & Kelly, 2021). To our knowledge, only two EMA studies in the field of SC exist so far assessed SC several times throughout the day, finding similar benefits of momentary SC. When assessed several times a day, Thøgersen-Ntoumani et al. (2017) found that, in moments when female individuals engaged in more appearance-related SC than usual, they were less socially anxious about their appearance, felt less drive for thinness, and were less dissatisfied with their bodies. Another recent study by Thøgersen-Ntoumani et al. (2021), which recruited overweight adults pursuing a weight loss plan, assessed SC in response to potential dietary lapses twice per day over 2 weeks and found more favorable reactions to a lapse in moments of higher SC. While these studies recruited mostly female participants and focused on the associations between SC and eating behavior or body image, they nevertheless show that SC may fluctuate throughout the day or week and that these fluctuations have important implications for concurrent well-being-related outcomes. Taken together, we can conclude that SC may not only benefit our well-being on the trait level (i.e., when we are a more self-compassionate person in general), but also on the state level (i.e., when we are treating ourselves with more SC in a certain moment).

An individual’s well-being varies constantly over time, often indicated by momentary levels of affect. It has been proven in multiple past studies that our affect is subject to constant fluctuations as a response to internal or external events or emotion regulation processes (e.g., Koval et al., 2015; Kuppens et al., 2010) and that these affective dynamics represent important indicators for psychopathology and well-being (see Houben et al., 2015, for a meta-analysis). Two frequently examined measures of affective dynamics are affective stress reactivity and emotional inertia, which may both be beneficially influenced by momentary SC.

Stress reactivity describes how strongly an individual affectively reacts to stressful events (Bolger & Zuckerman, 1995). In the context of affective dynamics, daily hassles are especially relevant, as these stressful events occur frequently in our everyday lives. For most of us, daily hassles are associated with subsequent decreases in well-being throughout the day (e.g., Mey et al., 2020). In their negative impact on health and well-being, daily hassles even seem to exceed the relevance of major life events (e.g., DeLongis et al., 1982). SC may positively influence how we cope with daily hassles in a certain moment. When we are more mindful of the daily hassle, instead of over-identifying with it, we may keep a more balanced perspective of the circumstances and how we relate to them (Desbordes et al., 2014). Keeping the common humanity of these everyday stressful events in mind may help us seeing stressful events simply as a normal part of our lives instead of a proof of our own incompetence (Neff, 2003b). The resulting feelings of connectedness may protect us from aggravating the personal extent of our suffering or from self-pity (Neff, 2003b), which has been associated with dysfunctional stress responses (Stöber, 2003). Treating ourselves with kindness after having experienced a daily hassle instead of condemning ourselves may help ourselves overcome the stressful events more quickly (Neff, 2003b). All in all, engaging in more SC in a certain moment in daily life may enable us to cope with stressful events more effectively and maintain well-being in the face of stress. This notion is supported by past studies, as outlined below.

Not only has trait and daily SC been associated with lower levels of perceived stress in general (e.g., Krieger et al., 2015; Li et al., 2020), but it also buffered the impact of stress on indicators of well-being such as depression, anxiety, and NA (Krieger et al., 2015; Stutts et al., 2018). Highly self-compassionate individuals seem to have better strategies at hand to cope with adverse events. For example, both healthy and depressed or anxious individuals with higher trait SC tend to apply more adaptive emotion-regulation strategies, such as more acceptance (Bakker et al., 2019), and less maladaptive strategies, such as denial or rumination (Bakker et al., 2019; Neff et al., 2005). A recent meta-analysis by Ewert et al. (2021) found moderate to strong overall correlations between trait SC and adaptive coping (*r* = 0.31) and maladaptive coping (*r* =  − 0.50). In sum, these findings show that momentary SC fosters a healthy response to adverse events.

A healthy response to adverse events is also characterized by the ability to quickly recover and detach from the negative affective states caused by these events, once they are over, instead of lingering in them for a longer time. Lingering in these past affective states may indicate a “resistance to emotional change” (Kuppens et al., 2010, p. 985), also called emotional inertia, which is the extent to which affective states are carried over from one moment to the next (e.g., Koval & Kuppens, 2012). High emotional inertia of both PA and NA demonstrates an impaired ability to flexibly regulate one’s emotions in response to changing situational requirements, thus depriving emotions from its adaptive purpose (Koval et al., 2015; Kuppens et al., 2010). Accordingly, high emotional inertia is connected to mental disorders such as depression (Kuppens et al., 2010) as well as to lower overall well-being, with NA inertia showing stronger and more consistent results than PA inertia (Houben et al., 2015; Koval et al., 2015). Even though no study has investigated how SC relates to emotional inertia, one can speculate that momentary SC may facilitate detachment from past affective states. Treating oneself with kindness rather than self-criticism and acknowledging the common humanity of one’s affective experiences in a certain moment in daily life might give oneself the emotional support and comfort needed to successfully overcome difficult emotional experiences and support individuals to perceive momentary unpleasant emotions as a natural part of our human life instead of feeling isolated and flawed for experiencing them (Neff, 2003b).

Furthermore, being self-compassionate involves being mindful towards outer and inner events. By adopting an accepting attitude and developing equanimity towards one’s present-moment experiences when being mindful, one may generate the ability to detach from past positive and negative affective states and regulate them more flexibly (Desbordes et al., 2014). Accordingly, mindfulness has been linked to lower emotional inertia, even though findings are not consistent. *Trait* mindfulness has been linked to lower levels of (low arousal) NA inertia; i.e., more mindful individuals lingered less in negative affective mood states, such as feeling depressed or sad. No connection was found to PA inertia (Keng & Tong, 2016; Rowland et al., 2020). Interestingly, *momentary* mindfulness was not related to NA inertia, but was found to be positively associated with low arousal PA inertia (i.e., being relaxed and satisfied; Rowland et al., 2020). Even though higher affective inertia in general had been associated with lower well-being (e.g., Houben et al., 2015), the authors argued that this persistent low arousal PA in moments of higher mindfulness could nevertheless be beneficial for an individual’s well-being. They built their argument on past research. Finding an upward spiral between state mindfulness and PA, in which these two processes mutually enhanced each other from one moment to the next (Gotink et al., 2016; Rowland et al., 2020). Taken together, even though the evidence on mindfulness and inertia is inconsistent, the theoretical considerations suggest that SC may facilitate a more flexible emotional response, minimizing both PA and NA inertia.

SC is typically conceptualized as the average score of all six subscales of the Self-Compassion Scale (SCS; Neff, 2003a) with each subscale representing one of the six components of SC. Evidence has been provided that this approach adequately reflects SC (e.g., Neff et al., 2019). However, some researchers have queried the validity of the SCS total score (e.g., López et al., 2015; Muris et al., 2021). Instead of confirming one general factor reflecting overall SC, an increasing number of factor-analytical studies have found evidence for two factors (for an overview, see Muris & Otgaar, 2020, p. 1474): one factor encompasses the positive components of SC (self-kindness, common humanity, and mindfulness), referred to as SC-Pos in the following. The other factor entails the negative components of SC (over-identification, isolation, and self-judgment, not reversed coded), which we will refer to as SC-Neg from now on. It has further been argued that the items of SC-Neg are confounded with psychological malfunctioning instead of simply reflecting the absence of SC (e.g., Muris et al., 2021). Consequently, the relationship between SC and psychopathology may be inflated (e.g., Muris et al., 2018, 2021). Thus, it has been suggested to not only rely on the total score of SC, but rather distinguish between SC-Pos and SC-Neg in order to properly map the true protective nature of SC (e.g., Brenner et al., 2017; López et al., 2015).

As one may expect, SC-Pos was positively related to indicators of well-being on the trait level, while SC-Neg showed negative correlations (e.g., Brenner et al., 2018; Chio et al., 2021). However, SC-Pos and SC-Neg differed regarding the strength of these associations, supporting claims for their distinction: SC-Neg (compared to SC-Pos) was relatively more strongly related to negative indicators of well-being, such as psychological distress (Chio et al., 2021), depressive symptoms, negative affect (López et al., 2016, 2018), or rumination and neuroticism (López et al., 2015). By contrast, SC-Pos showed greater effect sizes with mental well-being (Chio et al., 2021) or PA (e.g., López et al., 2015) compared to SC-Neg. Regarding their association with stress, SC-Pos correlated negatively with perceived stress while SC-Neg was positively related (Eriksson et al., 2018; López et al., 2015). Furthermore, SC-Pos was associated with more positive and less negative cognitive reactions to daily life problems as well as more adaptive coping in adolescents whereas the opposite pattern was identified for SC-Neg (Muris et al., 2019). These results suggest that SC-Pos may be associated with more favorable reactions to stress and, thus, potentially a lower stress reactivity, while SC-Neg may be connected to a higher stress reactivity. To our knowledge, no study has investigated the association between SC-Pos and SC-Neg and emotional inertia.

The present study aimed to examine how momentary SC in daily life relates to momentary well-being (as indicated by momentary affect), affective inertia, and stress reactivity in the same moment, assessing these constructs several times daily within an EMA design. In doing so, we intended to extend past studies regarding the following points: First, most studies on SC and well-being have understood SC as a trait (e.g., Krieger et al., 2015; Neff, 2003a), thereby neglecting important within-person fluctuations. Second, studies assessing SC as a state at one or two time-points in the laboratory (e.g., Kirschner et al., 2019) were not able to capture the dynamic interplay between SC, stressful events, and affect in real-life contexts, possibly dampening the ecological validity of findings (Shiffman et al., 2008). Third, the existing daily diary or EMA studies assessing SC daily or several times daily have not focused on the affective dynamics of emotional inertia and stress reactivity (e.g., Kelly & Stephen, 2016; Thøgersen-Ntoumani et al., 2017), which represent important proxies for well-being. Additionally, these studies have included mostly female participants, not being able to generalize results to all genders. To this end, we hypothesized that higher SC in a given moment would be associated with lower concurrent momentary NA (Hypothesis 1a) and higher momentary PA (Hypothesis 1b). Furthermore, we expected that higher SC in a given moment would be related to lower NA inertia (Hypothesis 2a) and lower PA inertia (Hypothesis 2b). Lastly, we hypothesized that higher SC in a given moment in daily life would be associated with a lower stress reactivity in terms of lower NA increases (Hypothesis 3a) and lower PA decreases (Hypothesis 3b) following the experience of daily hassles. We did not only examine the above hypotheses regarding the total score for SC, but also distinguished between the SC-Pos and SC-Neg to allow for a more nuanced and possibly less confounded evaluation of the benefits of SC for well-being, stress reactivity, and emotional inertia.

## Method

### Participants

This paper is based on data from the LifeStress study, a large longitudinal EMA project aiming to investigate the interrelations between daily hassles, resilience, and cardiovascular parameters in daily life. The study was implemented within two large longitudinal studies on resilience factors conducted within the Leibniz-Institute for Resilience Research and the University Medical Center in Mainz (for a more detailed description of the two studies, see Kalisch et al., 2020). Each participant took part in up to four 7-day EMA phases approximately every 6 months with data collection between 07/2018 and 11/2022 (recruitment for the first EMA phase completed in 02/2021). Items on SC had been added during the course of the study in 08/2019. We only analyzed the first complete wave of each participant which included items on SC. Data collection for this project took place between 08/2019 and 02/2021. In the following, only the method of the LifeStress study relevant for this paper will be presented.

Participants were non-clinical individuals from the general population, recruited via mails, letters, and flyers within the two parent studies mentioned above. In total, data from 119 participants was eligible for analyses. The mean age was 31.30 years (*SD* = 9.22 years). Sixty-four participants (53.78%) identified as female, 55 (46.22%) as male, and none as diverse. Regarding the highest completed level of education, two participants were still enrolled in high school, three participants had completed a basic secondary school (“Realschulabschluss”), 46 participants had finished high school with a general qualification for university entrance (“Abitur”), 19 had completed a professional training (“Ausbildung”), and 47 had finished university. Among all participants, 79 participants were currently part- or full-time (self-)employed, 52 were currently students or completing a professional training, one person was unemployed, and one person was on parental leave (multiple entries were possible). To be included in the LifeStress study, interested participants had to meet the following inclusion criteria at study entrance: (1) at least 18 years of age, (2) sufficient command of German to follow the instructions and to complete questionnaires, (3) previous experience in using smartphones, (4) no planned major deviation from the usual daily routine during the EMA phase (e.g., vacation), (5) no consumption of illegal drugs or large quantities of alcohol, and (6) no self-reported mental disorders. Additionally, all participants had been screened for mental disorders in structured clinical interviews (Mini International Neuropsychiatric Interview; Sheehan et al., 1998) as part of inclusion in the two larger studies. Current state of self-reported mental health was checked during the baseline and final sessions of the EMA phase of the LifeStress study with the General Health Questionnaire-28 (GHQ; Goldberg et al., 1997). The average GHQ score (*M* = 16.04, *SD* = 7.64) demonstrated an overall healthy sample. During the EMA phase, two individuals dropped out due to a lack of time and two due to problems with the cardiovascular measurement equipment of the LifeStress study. No participant indicated serious measurement effects of the EMA methodology during the final session. We excluded participants with a compliance rate below 50%, which was the case for one participant. Thus, the final sample comprised *n* = 114. In total, 4784 signals were available for data analysis. Of these 4784 signals, 4330 signals were answered completely. Overall, compliance was high with a mean of 90.50% (*SD* = 10.12%) completely answered signals per person.

### Procedures

**Fig. 1:**
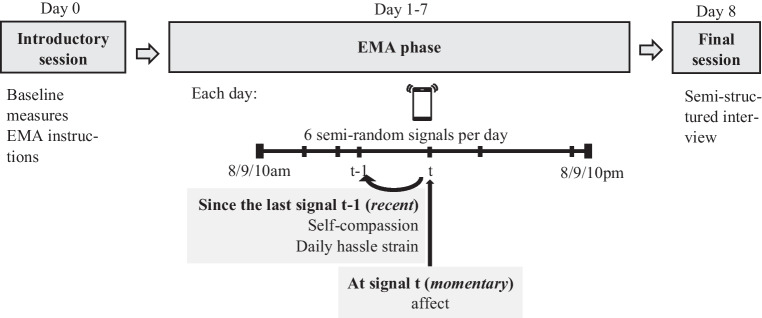
Overview of the study procedure

Figure 1 displays an overview of the study procedure. Before participation, participants provided informed consent. Participants meeting the inclusion criteria were invited to an introductory session in the laboratory and completed baseline measures. They were equipped with study smartphones (model Motorola Moto E; Chicago, IL) and received detailed instructions on how to complete the EMA signals. The 7-day EMA phase started on the day following the baseline session. Within a timeframe of 12 hr each day, starting at 8, 9, or 10am depending on the participant’s preference, participants received six semi-random signals on their study smartphone asking participants to indicate momentary affect as well as SC and occurred daily hassles since the last signal. Besides a minimum default time of 60 min between signals (*M* = 121.27 min, *SD* = 38.44), signals were randomly generated. Each signal had to be completed within 30 min with two reminders every 10 min. We employed the EMA software and App movisensXS (versions 1.4.8–1.5.18) to implement the EMA protocol on the smartphones. A final session in the laboratory or per telephone (during the COVID-19 pandemic) was scheduled for the day following the EMA phase, where participants underwent a semi-structured interview to obtain information about measurement effects of the EMA methodology. For their participation in the EMA phase (which included further measurements not mentioned in this paper), participants received a staggered financial compensation of up to 95 Euros, depending on their individual level of compliance. Each completely answered signal increased the amount of compensation. Additionally, participants could collect bonuses for compliance rates over 80% and 90%. Daily automated e-mails were sent out to inform about current signal compliance and the expected compensation.

### Measures

Each EMA signal comprised the following measures.

#### Positive and Negative Affect

Based on the affective circumplex model of emotions (Russell, 2003), participants rated how happy, anxious, satisfied, excited, sad, relaxed, depressed, and angry they felt at the moment on a slider style visual analog scale from 0 (*not at all*) to 100 (*very much*; Kuppens et al., 2010). The mean momentary PA was calculated by averaging the items happy, satisfied, excited, and relaxed. The mean momentary NA comprised the average of the remaining four items (Kuppens et al., 2010). We calculated McDonald’s omega as a measure for within- and between-person internal consistency (Hayes & Coutts, 2020). For within-person consistency, McDonald’s omega was *ω* = 0.69 for NA and 0.78 for PA. Regarding between-person reliability, we found a McDonald omega of *ω* = 0.92 for NA and *ω* = 0.86 for PA.

#### Self-compassion

As no validated state measure for SC had been available at the start of the study (the State Self-Compassion Scale was published by Neff et al., 2021, only recently), we assessed SC with six self-constructed items (Table 1). For item construction, we selected one item of each subscale of the short form (Raes et al., 2011) of the SCS (Neff, 2003a) and translated them into German, thereby orienting our wording on the respective items of the validated German version of the long form of the SCS (Hupfeld & Ruffieux, 2011). We slightly changed the wording to comply with assessment within an EMA protocol (e.g., shorter phrases, focus on the time interval since the last signal instead of general moments of suffering). Each item represented one of the six subscales of the trait SCS (self-kindness, self-judgment, common humanity, isolation, mindfulness, and over-identification). Participants indicated to which extent they treated themselves (un-)compassionately since the last signal on a slider style visual analog scale from 0 (*does not apply at all*) to 100 (*applies completely*). We calculated an overall SC score as the mean of all items after reversing the items for over-identification, isolation, and self-judgment (total score). We additionally computed separate scores for SC-Pos (mean of the items for self-kindness, common humanity, and mindfulness) and SC-Neg (mean of the unreversed items for self-judgment, isolation, and over-identification). Thus, higher SC-Pos scores indicate a more self-compassionate, kinder response while higher SC-Neg scores indicate a less self-compassionate, harsher response. In this study, within-person internal consistency of the six items amounted to a McDonald omega of *ω* = 0.46. Internal consistency of SC-Pos and SC-Neg items was slightly higher with 0.54 (SC-Pos) and 0.56 (SC-Neg). All scores indicate fair reliability (Shrout, 1998). Between-person reliability yielded higher parameters with a McDonald omega of *ω* = 0.61 for the complete six-item scale and *ω* = 0.87 (SC-Pos) and *ω* = 0.90 (SC-Neg). The improvement of the within- and between-person reliability scores for SC-Pos and SC-Neg compared to the full scale, as well as the low within- and between-person correlation between SC-Pos and SC-Neg (Table 2), support the notion of separating the positive components from the negative components when measuring SC. The person means of the scale exhibited medium to large correlations (Cohen, 1988) or “relatively large” correlations (Gignac & Szodorai, 2016) with the respective scores derived with the trait SCS (Neff, 2003a), administered during the baseline session (SC-Total score: *r* = 0.55, SC-Pos: *r* = 0.52, SC-Neg: *r* = 0.50), supporting the construct validity of the scale.

**Table 1:** Items assessing self-compassion (English translation and original German version) as well as respondent and scoring instructions

Subscore	Dimension	Item (English translation)	Item (original German version)
		Please indicate to which extent the following statements apply to you	Bitte geben Sie nun an, inwieweit die folgenden Aussagen auf Sie zutreffen
		Since the last signal…	Seit dem letzten Signal…
SC-Neg	Over-identification	“…I have mainly paid attention to everything that has bothered me about myself.”	“… habe ich hauptsächlich auf das geachtet, was mich an mir gestört hat.”
SC-Pos	Self-kindness	“…I have tried to be understanding and patient towards myself.”	“…habe ich versucht, mit mir selbst verständnisvoll und geduldig umzugehen.”
SC-Neg	Isolation	“…I have thought that most other people are probably happier at the moment than I am.”	“…habe ich gedacht, dass die meisten anderen Menschen wahrscheinlich gerade glücklicher sind als ich.”
SC-Pos	Common humanity	“…I have thought that it is human to make mistakes.”	“…habe ich gedacht, dass es menschlich ist, Fehler zu machen.”
SC-Pos	Mindfulness	“…I have tried to take a balanced view of things.”	“ …habe ich versucht, die Dinge nüchtern zu betrachten.”
SC-Neg	Self-judgment	“…I have condemned my own faults and weaknesses.”	“…habe ich meine eigenen Fehler und Schwächen verurteilt.”

*Note*. Response scale: visual analog scale from 0 (*does not apply at all*/*trifft gar nicht zu*) to 100 (*applies completely*/*trifft voll zu*). Scoring instruction: the total SC score was computed as the mean of all items after reversing the items for over-identification, isolation, and self-judgment. SC-Pos was computed as the mean of the items for self-kindness, common humanity, and mindfulness. SC-Neg was computed as the mean of the unreversed items for self-judgment, isolation, and over-identification

**Table 2:** Descriptive results (mean, standard deviation, range), ICCs, and within- and between-person correlations of the variables (*n* = 114)

	Computation	Possible range	Actual range	*M* (*SD*)	ICC	Within- and between-person correlations						
						1	2	3	4	5	6	7
1. SC (total score)	Mean of 6 items^a^	0–100	16.83–100	70.66 (13.85)	0.64	–	0.79***	− 0.61***	− 0.28***	0.29***	− 0.10***	− 0.01
2. SC-Pos	Mean of 3 items^a^	0–100	0–100	57.32 (22.67)	0.72	0.82***	–	− 0.09***	− 0.11***	0.18***	0.02	0.07***
3. SC-Neg	Mean of 3 items^a^	0–100	0–87.33	16.00 (16.29)	0.59	− 0.50***	0.08	–	0.36***	− 0.26***	0.19***	0.12***
4. NA	Mean of 4 items^a^	0–100	0–86.25	11.14 (12.67)	0.43	− 0.43***	− 0.03	0.70***	–	− 0.46***	0.33***	0.19***
5. PA	Mean of 4 items^a^	0–100	0.5–100	56.35 (17.37)	0.45	0.43***	0.46***	− 0.07	− 0.24**	–	− 0.33***	− 0.19***
6. Daily hassle strain	Total of strain ratings of 0–58 reported daily hassles^b^	0–232	0–44	2.02 (3.48)	0.41	− 0.10	0.03	0.21*	0.25**	− 0.10	–	0.80***
7. Daily hassle number	Total of 0–58 reported daily hassles	0–58	0–22	1.52 (2.12)	0.51	0.003	0.05	0.08	0.12	− 0.01	0.90***	–

*Note*. *SC*, self-compassion; *SC-Pos*, positive components of self-compassion (self-kindness, mindfulness, common humanity); *SC-Neg*, negative components of self-compassion (self-judgment, over-identification, isolation); *NA*, negative affect; *PA*, positive affect; *ICC*, intra-class correlation. Displayed statistics are based on the unstandardized variables (except for within-person correlations, which are based on the within-person standardized values). Within-person correlations are displayed above the diagonal, between-person correlations below the diagonal. ^a^Answering scale of each item 0–100. ^b^Answering scale of each strain rating: 0–4

^*^p < 0.05, **p < 0.01, ***p < 0.001

#### Daily Hassle Strain

Daily hassles were assessed with an EMA version of the Mainz Inventory of Microstressors (MIMIS; Chmitorz et al., 2020). It consists of a list of 58 potential daily hassles covering different aspects of daily life (e.g., work, family, friends, monetary aspects). Individuals selected each daily hassle, which had occurred since the last signal, from the list. For each selected daily hassle, they subsequently rated the perceived daily hassle strain on a 5-point Likert scale from 0 (*not at all straining*) to 4 (*very straining*). We calculated the daily hassle strain for each signal as the sum of all strain ratings in one signal. If participants had not experienced any daily hassle, they could choose the option “none of the above events has occurred since the last signal” at the end of the list. In these cases, the daily hassle strain was coded as zero. Evidence for the reliability of the MIMIS has been provided in a past study (Chmitorz et al., 2020), demonstrating high correlations regarding daily hassle number and mean strain between the EMA version of the MIMIS and respective end-of-day and end-of-week versions.

### Data Analyses

All analyses were performed in Stata 15 (Stata Corporation, College Station, TX, USA). We estimated two-level models with random intercepts and slopes to consider the hierarchical data structure of daily signals (level 1) nested within participants (level 2), thereby accounting for both within-person variation and between-person differences in this data. If possible, an unstructured covariance matrix was requested in all models on level 2 to allow for random covariation between random intercepts and slopes (Robson & Pevalin, 2016). All predictors were added as random effects on the participant level, allowing the slopes to vary randomly between individuals (Robson & Pevalin, 2016). Furthermore, instead of implementing within-person centering, we within-person standardized all level-1 predictors and outcomes by taking the difference between the score of an observation and the person mean of this variable and dividing it by the person standard deviation of this variable. This approach facilitates the interpretation of the effect sizes and better controls for between-person differences in the estimates of the slopes compared to within-person centering (Wang et al., 2019). We suppressed the constant in the random effects part of the model, as the standardized constant would equal zero, ruling out possible covariations. From now on, all variables which were measured at signal* t* but referred to the interval between time-points *t-*1 and* t* are designated as *recent*. Variables assessed at signal* t* referring to the current moment of the signal* t* are named *momentary*. *Prior* identifies a variable at the prior signal *t-*1 and is conceptualized as a lagged variable. To prevent effects from the previous day to obscure results, the first signal of each day was replaced with a missing value for the lagged variables. The EMA signal number (across the study) was included as a fixed effect to control for possible time trends. All models were controlled for recent daily hassle strain as well as prior PA (or prior NA, respectively) to account for differences attributable to these variables instead of recent SC.

To test the hypotheses, momentary affect was predicted using the equations below. Each model was carried out separately for the prediction of momentary PA and NA (equations are only presented for PA but apply analogously to NA). All models were first tested for the total score (recent SC, Models 1.1, 2.1, and 3.1). To test whether recent SC-Neg and SC-Pos were differently associated with affective dynamics, we repeated these models by replacing recent SC with recent SC-Neg and recent SC-Pos simultaneously in the same model, thereby controlling for the respective other subscores (Models 1.2, 2.2, and 3.2).

#### Model 1.1: Association Between SC and Affect (Hypotheses 1a and b)

Momentary *PA*_*ij*_ indicates the momentary PA of participant *i* at time-point *j*. ${\beta }_{0}$ to ${\beta }_{4}$ are the level-2 intercepts and show how the respective variable is associated with PA on average in the sample. Due to standardization of all variables (except signal number), they can be interpreted as standardized regression weights. ${u}_{1i}$ to ${u}_{3i}$ indicate the random effects, which allow the intercept and slopes to vary between participants. Thus, ${\beta }_{0}+ {u}_{0i}$ denotes the intercept reflecting the mean momentary PA for participant *i* across all signals assuming all other variables to be at the person mean. Accordingly, ${\beta }_{1}+ {u}_{1i}$, ${\beta }_{2}+ {u}_{2i}$, and ${\beta }_{3}+ {u}_{3i}$ describe the slopes for recent SC, recent daily hassle strain, prior PA, and signal number and represent the average effect of these variables on momentary PA for participant *i* across all signals. Error term ${\upepsilon }_{ij}$ reflects the within-person variability, which allows momentary PA at a given time-point to differ from the person mean.

1 $${\mathrm{Momentary\;} PA}_{ij}= {(\beta }_{0}+ {u}_{0i})+ {(\beta }_{1}+ {u}_{1i}) \mathrm{\;recent\;} SC+ {(\beta }_{2}+ {u}_{2i}) \mathrm{\;recent\;} \mathrm{\;daily} \mathrm{\;hassle} \mathrm{\;strain}+ {(\beta }_{3}+ {u}_{3i}) \mathrm{\;prior\;} PA+ {\beta }_{4} \mathrm{signal\;number}+{\upepsilon }_{ij}$$

#### Model 2.1: Moderation of Affective Inertia by SC (Hypotheses 2a and b)

Emotional inertia is operationalized as the autoregressive slope ${\beta }_{3}+ {u}_{3i}$ (e.g., Koval et al., 2015), which indicates how strongly a person’s prior affect is predictive of their momentary affect. Higher scores reflect higher inertia. Accordingly, the interaction term prior PA $\times$ recent SC represents changes in PA inertia depending on recent SC. ${\beta }_{5}+ {u}_{5i}$ stands for the respective slope.

2 $${\mathrm{Momentary\;} PA}_{ij}= {(\beta }_{0}+ {u}_{0i})+ {(\beta }_{1}+ {u}_{1i}) \mathrm{\;recent\;} SC+ {(\beta }_{2}+ {u}_{2i}) \mathrm{\;recent\;daily\;hassle\;strain}+ {(\beta }_{3}+ {u}_{3i}) \mathrm{\;prior\;} PA+ {\beta }_{4} \mathrm{signal\;number}+{(\beta }_{5}+ {u}_{5i}){ \mathrm{\;prior\;} PA\times \mathrm{\;recent\;} SC + \epsilon }_{ij}$$

#### Model 3.1: Moderation of Stress Reactivity by SC (Hypotheses 3a and b)

Stress reactivity is conceptualized as the slope ${\beta }_{2}+ {u}_{2i}$, reflecting to which extent a person’s momentary affect is related to their experienced recent daily hassle strain. Higher PA stress reactivity (i.e., a stronger decrease in PA decreases following the experience of daily hassles) is indicated by higher negative scores. Higher NA stress reactivity (stronger increase in NA following the experience of daily hassles) is reflected by higher positive scores. The interaction term recent daily hassle strain $\times$ recent SC reflects changes in stress reactivity depending on recent SC. Again, ${\beta }_{5}+ {u}_{5i}$ stands for the respective slope.

3 $${\mathrm{Momentary\;} PA}_{ij}= {(\beta }_{0}+ {u}_{0i})+ {(\beta }_{1}+ {u}_{1i}) \mathrm{\;recent\;} SC+ {(\beta }_{2}+ {u}_{2i}) \mathrm{\;recent\;daily\;hassle\;strain}+ {(\beta }_{3}+ {u}_{3i}) \mathrm{\;prior\;} PA+ {\beta }_{4} \mathrm{\;signal\;number}+{(\beta }_{5}+ {u}_{5i}) \mathrm{\;r}{\mathrm{ecent\;daily\;hassle\;strain}\times \mathrm{\;recent\;SC} + \epsilon }_{ij}$$

#### Exploratory Analysis on the Temporal Associations Between SC and Affect

While Model 1.1 may confirm the associations between recent SC and momentary affect, it does not allow for assumptions regarding the direction of the effect. We cannot answer whether higher recent SC leads to higher momentary PA and lower NA, or whether, vice versa, a more positive affective state of higher PA and lower NA facilitates a more self-compassionate response. Even though we cannot state causality, we aimed to shed more light onto the temporal associations between SC and affect. For this purpose, we conducted an additional exploratory analysis, considering the different time frames individuals are asked to refer to when answering the questions regarding SC and affect. While affect at signal *t* refers to the very moment of answering the signal, SC reported at signal *t* encompasses the complete time frame since the last signal *t*-1, implying a temporal order: the “act” of SC most likely took place before the experience of the reported momentary affect at signal *t*. By contrast, the momentary affect reported at the prior signal *t*-1 precedes the act of SC between *t*-1 and *t* (reported at *t*). Thus, we conducted two models with prior PA (or NA, respectively) predicting recent SC, controlling for prior SC, recent daily hassle strain, and signal number (all variables also added as random effects). Then, we compared the effect sizes of prior PA (or prior NA, respectively) predicting recent SC in this model to the effect sizes of recent SC predicting momentary PA (or NA, respectively, Models 1.1). Even though we still cannot infer causality, we may draw conclusions about the temporal order of SC and affect, depending on which model reveals higher effect sizes, i.e., which temporal association was stronger.

## Results

Descriptive statistics as well as within- and between-person correlations of the main variables can be found in Table 2. Results of the multilevel models are presented in Tables 3 and 4. Corresponding path diagrams can be found in the supplement (Supplementary Fig. S1).

**Table 3:** Multilevel models to examine affective dynamics of SC (total score)

	Momentary NA			Momentary PA		
Within-individuals fixed effects	Estimate	*SE*	[95% CI]	Estimate	*SE*	[95% CI]
Association between affect and SC (Models 1.1)						
Recent SC (total score)	− 0.228***	0.023	[− 0.273; − 0.184]	0.223***	0.022	[0.181; 0.266]
Recent daily hassle strain	0.311***	0.023	[0.265; 0.357]	− 0.293***	0.022	[− 0.336; − 0.250]
Prior NA (prior PA respectively)	0.140***	0.019	[0.103; 0.178]	0.276***	0.018	[0.241; 0.312]
Signal number	0.001	0.001	[− 0.001; 0.004]	− 0.008***	0.001	[− 0.010; − 0.006]
Affective inertia (Models 2.1) ^a^						
Recent SC $\times$ prior NA (prior PA respectively)	− 0.021	0.016	[− 0.053; 0.011]	− 0.016	0.014	[− 0.044; 0.013]
Stress reactivity (Models 3.1) ^a^						
Recent SC $\times$ recent daily hassle strain	− 0.128***	0.021	[− 0.170; − 0.086]	0.050**	0.016	[0.018; 0.082]

*Note*. *SC*, self-compassion; *NA*, negative affect; *PA*, positive affect; *CI*, confidence interval. 3325 observations, 114 individuals. All level-1 variables were within-person standardized (except signal number). Fixed main effects parameters in Models 2.1 and 3.1 are not displayed for better clarity, as they were almost identical to those in Models 1.1. All *p* values are two-tailed. ^**a**^An unstructured covariance matrix was not requested in Models 2.1 and 3.1 predicting PA to allow model convergence

^*^p < 0.05, **p < 0.01, ***p < 0.001

**Table 4:** Multilevel models to examine affective dynamics of SC-Pos and SC-Neg

	Momentary NA			Momentary PA		
Within-individuals fixed effects	Estimate	*SE*	[95% CI]	Estimate	*SE*	[95% CI]
Association between affect and SC (Models 1.2) ^a^						
Recent SC-Pos	− 0.085***	0.019	[− 0.123; − 0.047]	0.149***	0.022	[0.107; 0.192]
Recent SC-Neg	0.265***	0.022	[0.222; 0.308]	− 0.156***	0.018	[− 0.190; − 0.121]
Recent daily hassle strain	0.286***	0.023	[0.240; 0.331]	− 0.291***	0.021	[− 0.333; − 0.249]
Prior NA (prior PA respectively)	0.124***	0.019	[0.088; 0.161]	0.268***	0.018	[0.233; 0.303]
Signal number	0.001	0.001	[− 0.001; 0.003]	− 0.009***	0.001	[− 0.011; − 0.006]
Affective inertia (Models 2.2) ^a^						
Recent SC-Pos $\times$ prior NA (prior PA respectively)	− 0.016	0.016	[− 0.047; 0.015]	− 0.016	0.015	[− 0.047; 0.014]
Recent SC-Neg $\times$ prior NA **(**prior PA respectively)	0.019	0.016	[− 0.013; 0.050]	0.007	0.015	[− 0.023; 0.037]
Stress reactivity (Models 3.2) ^a^						
Recent SC-Pos $\times$ recent daily hassle strain	− 0.094***	0.022	[− 0.137; − 0.051]	0.029	0.020	[− 0.010; 0.069]
Recent SC-Neg $\times$ recent daily hassle strain	0.056*	0.023	[0.010; 0.101]	− 0.020	0.015	[− 0.050; 0.011]

*Note. SC-Pos*, positive components of self-compassion (self-kindness, mindfulness, common humanity); *SC-Neg*, negative components of self-compassion (self-judgment, over-identification, isolation); *NA*, negative affect; *PA*, positive affect; *CI*, confidence interval; 3325 observations, 114 individuals. All level-1 variables were within-person standardized (except signal number). Fixed main effects parameters in Models 2.2 and 3.2 are not displayed for better clarity, as they were almost identical to those in Models 1.1. All *p* values are two-tailed. ^**a**^An unstructured covariance matrix was not requested in Model 1.2 predicting PA, Models 2.2 predicting PA and NA, and Models 3.2 predicting PA and NA to allow model convergence

^*^p < 0.05, **p < 0.01, ***p < 0.001

### Association Between SC and Affect (Hypotheses 1a and b)

As hypothesized, recent SC was significantly associated with increased momentary PA and decreased momentary NA (Table 3, Model 1.1). Regarding the subscores of SC, we found recent SC-Pos to be related to increased momentary PA and decreased momentary NA, while recent SC-Neg revealed a significant association with decreased momentary PA and increased momentary NA (Table 4, Model 1.2). While the effect sizes of recent SC-Pos and SC-Neg were similar in size for momentary PA, the parameter was higher for SC-Neg (compared to SC-Pos) when predicting NA (considering the non-overlap of the absolute values of the 95% confidence intervals of SC-Pos and SC-Neg).

### Moderation of Affective Inertia by SC (Hypotheses 2a and b)

We did not find significant associations between any of the SC measures and PA or NA inertia. Thus, the extent to which individuals acted in a (un-)compassionate way toward themselves did not seem to meaningfully alter their capacity to disengage from prior affective states.

### Moderation of Stress Reactivity by SC (Hypotheses 3a and b)

**Fig. 2:**
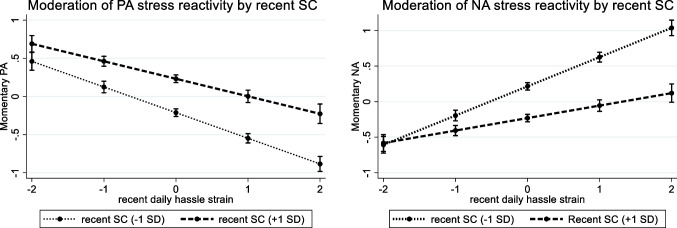
Moderation of PA and NA stress reactivity by SC (total score). *Note.* All variables are within-person standardized. 0 reflects the respective person mean, and other values show the respective standard deviation (SD) from the person mean. The graphs show the associations for 1 SD above and below the person mean in SC. Markers include the 95% confidence intervals

We found that recent SC significantly moderated PA and NA stress reactivity in the expected direction: when participants reported higher recent SC than usual, they experienced a lower stress reactivity, meaning that they reported lower decreases in PA and lower increases in NA after being confronted with daily hassles compared to times with lower levels of SC (Table 3, Model 3.1; see Fig. 2 for a visualization). The moderating effect of SC on NA stress reactivity was bigger than the effect on PA stress reactivity (based on the non-overlap of the absolute values of the 95% confidence intervals), suggesting that SC may particularly prevent individuals from experiencing increases in NA in the face of stress. Regarding SC-Pos and SC-Neg, we found both to be only associated with NA stress reactivity but not to significantly moderate PA stress reactivity. SC-Pos was associated with an attenuated NA stress reactivity, while SC-Neg (thus, scoring higher on over-identification, isolation, and self-judgment) was related to an increased NA stress reactivity (Table 4, Model 3.2). Thus, when individuals actively responded in a more self-compassionate, caring way and less harshly toward themselves than usual, they experienced lower increases in NA (but not lower PA decreases) when confronted with daily hassles compared to moments of lower SC.

#### Exploratory Analysis

In contrast to the effect sizes of recent SC predicting momentary PA/NA (Model 1.1), we found insignificant weak effect sizes for prior PA/NA predicting recent SC, even though the estimates for prior PA and NA almost reached significance (prior PA: *β* = 0.042, *SE* = 0.022; *p* = 0.054; CI: − 0.001; 0.084; prior NA: *β* =  − 0.038, *SE* = 0.020; *p* = 0.052; CI: − 0.077; 0.0004). The absolute values of the 95% confidence intervals did not overlap with the respective values of Model 1.1, suggesting that the associations between recent SC and momentary PA/NA were stronger than the association between prior PA/NA and recent SC. These results imply that SC was more responsible for subsequent affect but not vice versa.

## Discussion

While the benefits of trait SC for an individual’s overall well-being have been proven multiple times in past research (e.g., Zessin et al., 2015), little has been known about within-person relations between momentary SC and well-being in everyday life. The present study aimed at examining whether having recently acted self-compassionately was associated with higher PA and lower NA (both key indicators for well-being) in the next moment, and whether recent SC was related to the affective dynamics pattern of emotional inertia (which has been linked to lower well-being and mental health) and stress reactivity in daily life.

As hypothesized, we found that when individuals had responded more compassionately toward themselves (irrespectively of how self-compassionate they were as individuals), they reported more PA and less NA in the next moment. This result is in line with past between-person research demonstrating higher overall well-being (e.g., Zessin et al., 2015) as well as higher PA and lower NA in more self-compassionate individuals (e.g., Hupfeld & Ruffieux, 2011; Krieger et al., 2015). Additionally, our results go along with recent findings by Neff et al. (2021) of higher momentary SC to be associated with higher momentary PA and lower NA at the same time-point in the laboratory. We conducted an additional exploratory analysis to clarify the temporal associations between SC and affect, finding recent SC to predict subsequent affect more strongly rather than the other way around. While we cannot infer causality, these results suggest that SC may be more responsible for subsequent affect than vice versa. A longitudinal study by Krieger et al. (2016) backs up this notion, finding trait SC to predict depressive symptoms 6 months later, but not the other way around. All in all, our results emphasize once more the protective role of SC for an individual’s well-being and expand the trait-level results to the state level: The benefits of SC for well-being do not only result from being a more self-compassionate person but can also unfold in a given moment when someone treats themself with more compassion. Our results may offer implications for trait levels of SC as well. Repeatedly increasing one’s momentary SC may ultimately raise trait levels of SC, drawing from studies on mindfulness, which have shown that repeatedly increasing state mindfulness in brief meditations over several weeks led to heightened trait mindfulness at the end of the intervention (Kiken et al., 2015). Thus, while it may still not be easy to generate momentary SC during a challenging situation in daily life, treating oneself with compassion in a given moment may be a tool to help individuals to become more self-compassionate as a person, offering significant benefits for overall well-being (e.g., Zessin et al., 2015).

Regarding the distinction between SC-Pos and SC-Neg, we found higher SC-Pos in a certain moment to be associated with higher PA and lower NA. Higher SC-Neg (thus, scoring higher on over-identification, isolation, and self-judgment) corresponded to these findings, relating negatively to PA and positively to NA. Looking at the effect sizes reveals a more nuanced picture. Compared to SC-Pos, SC-Neg was relatively more strongly connected to NA while SC-Pos and SC-Neg were similarly connected to PA. This finding indicates that it is equally important for momentary PA to respond in a kinder and more compassionate way while at the same time behave in a less criticizing way toward the self. In contrast, a harsh, judgmental self-response seemed to be more connected to momentary NA compared to an explicit friendly, compassionate self-response. These results correspond to past studies finding the negative components of SC (compared to the positive ones) to be relatively more strongly connected to negative indicators of well-being such as psychological distress (Chio et al., 2021), depressive symptoms, negative affect (López et al., 2016, 2018), or perceived stress, rumination, and neuroticism (López et al., 2015). Similarly, Muris et al. (2018) and Muris et al. (2021) found that SC-Neg explained most of the variance in internalizing symptoms leaving only a small share for SC-Pos. While the similar effect sizes of SC-Neg and SC-Pos regarding PA found in this study correspond to Brenner et al. (2018), they contradict other studies finding SC-Pos (compared to SC-Neg) to correlate relatively more strongly to PA (e.g., López et al., 2016, 2018) and well-being (Chio et al., 2021). However, the recent meta-analysis by Chio et al. (2021) found differences in effect sizes to be small (albeit significant). The authors reasoned that the inclusion of the component common humanity, which exhibited the smallest effect sizes compared to the other components, may have weakened the overall predictability of SC-Pos.

Contrary to our expectations, SC seemed unrelated to both PA and NA inertia (regardless of its quantification as the total score, SC-Pos or SC-Neg). Treating oneself with more SC in a certain moment in daily life did not seem to change a person’s ability to affectively disengage from previous affective experiences. One explanation for this result may be the temporal resolution of the assessments in this study. Six daily assessments (thus, one assessment approximately every 2 hr) may have been too coarse to properly capture the effect of SC on emotional inertia, which may have occurred on a shorter timescale.

The apparently missing link between inertia and SC may reflect a “paradox” within the SC concept (Germer & Neff, 2019). On the one hand, SC generates “the desire to alleviate one’s suffering” (Neff, 2003b, p. 87), and thus wishing to relieve unpleasant emotions, while on the other hand, SC involves the acceptance of the current-moment experience, as represented by the mindfulness component (Neff, 2003b). Full acceptance, however, allows an individual to be with an unpleasant emotion just “as it is” without a need for change, creating the paradox. Misusing SC to solely “alleviate one’s suffering” without fully accepting one’s unpleasant feelings at the same time may be a subtle, but maladaptive form of resistance against negative emotions, which in turn may increase one’s suffering (Germer & Neff, 2019). However, fully accepting something unpleasant is not easy. Mindfulness research has shown that the skill of acceptance may require more time and training to develop than other mindfulness components, such as mere observational or attentional skills (Baer et al., 2012; Desbordes et al., 2014). This may also explain why only individuals with prior mindfulness meditation experience (but not novices) seemed to be able to effectively detach from past emotions when being more mindful (Rowland & Wenzel, 2020). Thus, the benefits of SC (and mindfulness) on affective detachment may only unfold after significant training and internalization of the importance to fully accept the present moment experience. Concluding from the general German public, most of our participants presumably were not acquainted with SC or mindfulness practices and thus may not have internalized the process of acceptance sufficiently, possibly explaining the missing link between SC and inertia.

According to our hypothesis, when individuals treated themselves with more SC than usual, they reported a lower NA and PA stress reactivity in terms of lower PA decreases and lower NA increases following the experience of daily hassles. Our results correspond to findings on the trait level showing higher trait SC to weaken the link between perceived stress and indicators of well-being such as depression, anxiety, or NA (Stutts et al., 2018) and extend them to the state level. SC may not only buffer the effect of stress on well-being when we are more self-compassionate as a person, but also when we treat ourselves with more SC in a stressful moment of daily life (no matter how self-compassionate we may be as individuals). The association between SC and stress reactivity may be explained by more adaptive emotion regulation strategies, as it has been found on the trait level (e.g., Bakker et al., 2019; Neff et al., 2005). In more detail, we found that SC may particularly prevent individuals from experiencing increases in NA in the face of stress, as the moderating effect of SC on NA following daily hassles was larger than the effect on PA. These findings are in line with past research, which consistently found trait SC to moderate the effect of stress on NA but reported either less consistent results regarding the moderation of stress on PA (Stutts et al., 2018) or no relation between SC and PA stress reactivity at all (Krieger et al., 2015). Thus, SC may support individuals to regain emotional balance in stressful situations by reducing negative stress reactivity, but not so much by explicitly fostering positive emotions. This notion corresponds to our findings regarding SC-Pos and SC-Neg, which were both only associated with NA stress reactivity but did not significantly moderate PA stress reactivity. SC-Pos was associated with an attenuated NA stress reactivity, and SC-Neg with an increased NA stress reactivity. Thus, one may conclude that treating oneself with compassion *or* refraining from harsh self-criticism by itself may not be enough to maintain positive emotions in the face of stress in a given moment. Rather, one may need to be mindful of both ways of self-responding at the same time.

### Limitations and Future Directions

The results of this study should be interpreted in the light of some limitations. First, the measure of momentary SC was an ad hoc measure devised for the purpose of this study, as no validated measure for momentary SC had been available at the beginning of the study. While the reliability parameters of the total scale as well as the SC-Pos and SC-Neg subscales appear to be low when evaluated with guidelines for trait measures, it has been proposed to employ more relaxed guidelines for within-person reliability (Nezlek, 2017). Furthermore, low omega coefficients for within-person scores may also reflect a sensitivity of the scale to measure state changes, indicating state validity (Medvedev et al., 2017). Nevertheless, the low reliability may have led to an underestimation of SC scores and an attenuation of the validity of our results. Furthermore, our scale did not explicitly focus on negative experiences or painful moments and rather assessed a broader self-compassionate attitude to allow for a more continuous and extensive assessment of SC. While our scale showed significant correlations with the validated trait version of the SCS, it may nevertheless have tapped into a more general concept of self-warmth or self-acceptance. Furthermore, the wording of the items representing SC-Pos may have additionally obscured results due to the inclusion of the verb “try” (mirroring the wording of the trait SCS items). While even trying to be kind and patient toward oneself may be an act of SC, our items do not offer information whether one actually managed to be kind toward the self. These different levels of “commitment” to the SC concept may be differentially associated with well-being, inertia, and stress reactivity. It may be advisable to replicate our results applying a validated measure for momentary SC, such as the recently published State Self-Compassion Scale by Neff et al. (2021). An additional limitation of our study is that we cannot conclude causal effects of SC with our results. Even though the reported SC in each signal refers to the time frame since the last signal, implying that SC took place before the experience of affect reported in each signal, we can only assume temporal associations but cannot infer causality. The results of the exploratory analysis on these temporal associations need to be interpreted with caution, as potential measurement effects, response biases, or third variables could have influenced the results. For example, simply the fact that recent SC and momentary affect were assessed at the same signal (vs. two subsequent signals as it is the case for prior affect and recent self-compassion) could have increased the association between these variables. Additionally, as mentioned earlier, the temporal resolution of assessments may have limited the ability of the study to adequately capture the dynamic interplay between SC and inertia. Future studies may increase the number of assessments per day to potentially uncover more subtle effects of SC on inertia. Moreover, the results of this study rely on self-report data, which naturally implies some limitations, such as recall or response biases (even though less pronounced due to the EMA methodology compared to laboratory-based studies; Shiffman et al., 2008). Lastly, we did not assess previous experience with the SC concept or SC meditation, which may be an important moderator of the effects of SC on well-being, as discussed earlier.

Future research on SC may benefit from applying an EMA approach. First, with its frequent assessments, the EMA methodology is well-suited to capture the within-person changes (Shiffman et al., 2008), which SC evidently displays. In this study, we found an intra-class correlation (ICC) of 0.64, which is similar to the ICCs reported by the small number of other studies assessing SC daily or several times daily (e.g., Breines et al., 2014; Katan & Kelly, 2021). This ICC indicates that a substantial amount of SC variance was attributable to within-person variance, suggesting that SC is, indeed, not only a stable personality trait but also a fluctuating state. Importantly, both in our study and past research, these fluctuating states covaried with markers for health and well-being (e.g., Breines et al., 2014; Li et al., 2020). This emphasizes once more the need to adequately capture and investigate the momentary process of SC fluctuations and their correlates.

Furthermore, the frequent measurements within an EMA protocol reduce retrospective biases, which may obscure recalls of past events and confound results (Shiffman et al., 2008). Additionally, EMA allows the data to be collected within participant’s everyday lives instead of in a laboratory. Thus, the data are collected where the application of SC is most relevant and meaningful, increasing the ecological validity and generalizability of the results to real-life experiences (Shiffman et al., 2008). Due to these benefits, EMA methodologies may draw a more comprehensive and nuanced picture of state SC, such as its short-term benefits or the short-term mechanisms by which SC may cause these benefits. EMA may also offer a high-quality design to investigate underrepresented areas of research on SC, which have recently been identified within a science-mapping analysis by Swami et al. (2021). Furthermore, so-called ecological momentary interventions may offer new opportunities to help individuals develop a more self-compassionate attitude in everyday life by implementing novel smartphone-based SC trainings.

## Supplementary Information

Below is the link to the electronic supplementary material.

Supplementary file1 (DOCX 63 KB)

## Data Availability

Data is available at the Open Science Framework (https://osf.io/pve7u/?view_only=0350de7ec3a5487190add0253aebdec2).
