# Supplementary material for: Be Kind to Yourself: the Implications of Momentary Self-Compassion for Affective Dynamics and Well-Being in Daily Life
Source: Mindfulness (N Y). 2023 Jan 7;14(3):622–36. doi: 10.1007/s12671-022-02050-y (PMC9823261; doi:10.1007/s12671-022-02050-y)
Supplement: Supplementary file 1 — Supplementary file1 (DOCX 63 KB) [file 12671_2022_2050_MOESM1_ESM.docx]

**SUPPLEMENT**


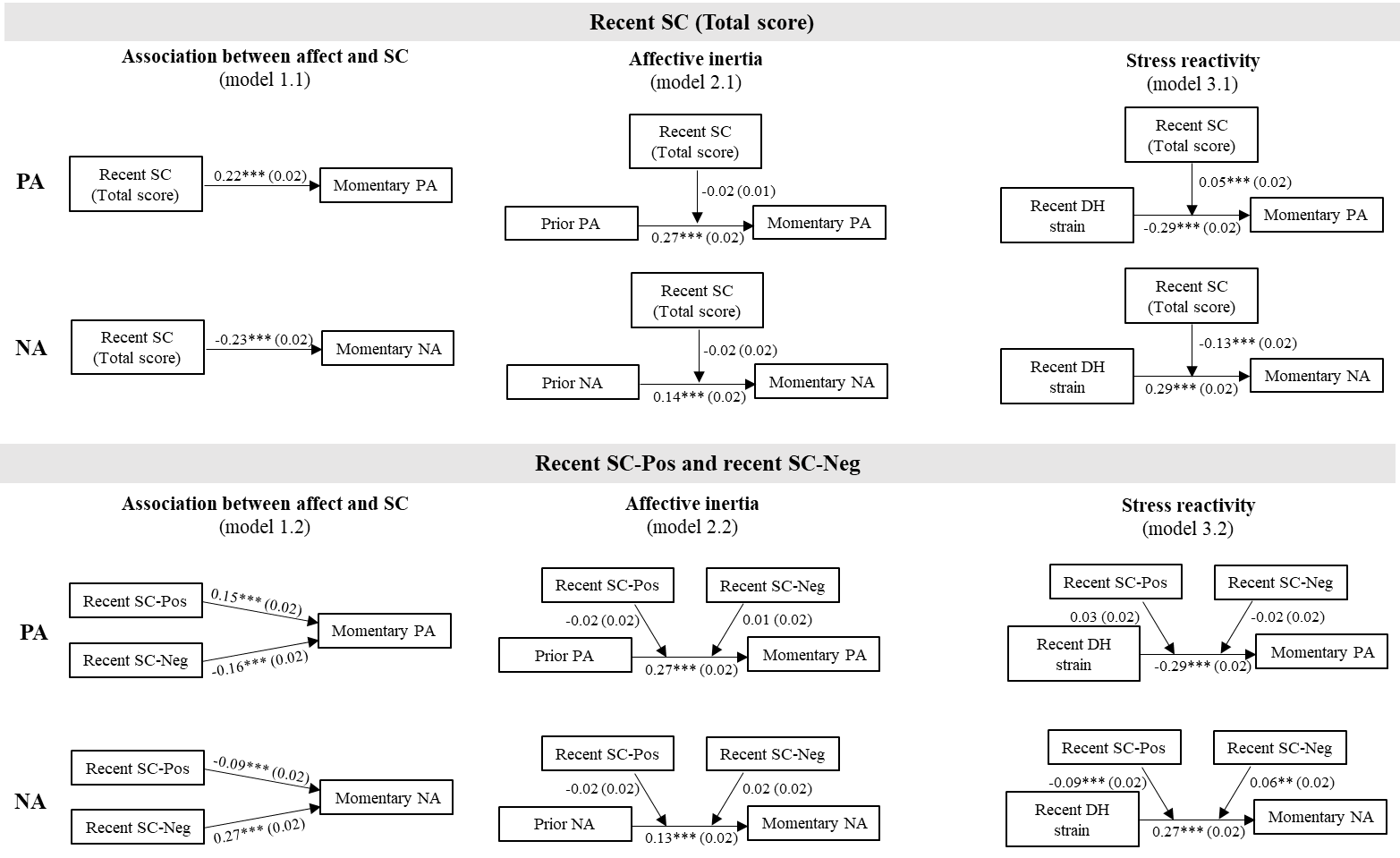
Figure S1. Path diagrams of the multilevel models.

*Note*. Displayed statistics are the estimates (standard errors in brackets) of the respective multilevel models (based on within-person standardized variables).

**p* < .05, ***p* < .01, ****p* < .001.
